# Circular RNAs: An Under-Recognized Part in Different Scenarios of Host–Parasite Interrelationships

**DOI:** 10.3390/pathogens15030307

**Published:** 2026-03-11

**Authors:** Mahmoud S. Sharaf, Dalia S. Ashour, Ahmad A. Othman

**Affiliations:** Medical Parasitology Department, Faculty of Medicine, Tanta University, Tanta 31527, Egypt; mahmoud.sharaf@med.tanta.edu.eg (M.S.S.); dalia.ashour@med.tanta.edu.eg (D.S.A.)

**Keywords:** circular RNA, non-coding RNA, circRNAs and parasites, host circRNAs, host–parasite interaction, *Plasmodium*, *Schistosomes*

## Abstract

Circular RNAs (circRNAs) are a special category of non-coding RNAs. The latter are known for their pivotal regulatory functions over many cellular processes. In biology, circRNAs exert regulatory functions on many physiological events, most likely through the regulation of gene expression. They are also implicated in a variety of health-related issues in medicine. Advances in molecular techniques and bioinformatics are expected to expand our knowledge of circRNAs, better characterizing their nature and functions. Remarkably, parasites elaborate their own repertoire of circRNAs to regulate different biological aspects. Meanwhile, they reshape the host circRNA landscape, allowing manipulation of different events of host–parasite interplay at molecular levels. We herein present an overview of the putative role of circRNAs in major parasitic infections of medical importance. Surprisingly, we underscore limited knowledge in this domain with many gaps and uncertainties. Scrutiny into the role of circRNAs in host–parasite dynamics could lead to the development of reliable diagnostic tools, or the discovery of novel therapeutic targets or vaccine candidates, for many parasitic infections.

## 1. Introduction

Non-coding RNAs (ncRNAs) are functional RNA molecules that do not encode proteins but play crucial roles in regulating gene expression and cellular functions. They are broadly classified based on their length into short and long ncRNAs (lncRNAs) [1,2].

Circular RNAs (circRNAs) are long, non-coding endogenous single-stranded closed-loop RNA molecules [3]. circRNAs were first reported as viroids, pathogens of higher plants, by Sanger and coworkers in 1976 [4], and were first detected in the cytoplasm of human HeLa cells by electron microscopy in 1979 [5]. Later, with the development of high-throughput RNA-sequencing and bioinformatics tools, scientists have found circRNAs in various species with high cell/tissue specificity [6,7]. CircRNAs are found in nearly all mammals [8], plants [9], parasites [10], and viruses [11].

In this review, we provide an overview of the general biology of circRNAs and the research findings about the role of these molecules during parasitic infections of medical importance, aiming to deepen our understanding of different aspects of host–parasite crosstalk. We shall also discuss the implications, limitations, and future perspectives of research on circRNAs in the field of human parasitology.

## 2. Life History of circRNAs: From Cradle to Grave

### 2.1. Biogenesis

Generally, pre-mRNA is transcribed from DNA by RNA polymerase II enzyme (Pol II). It contains introns and exons, followed by adding a methylguanosine cap to its 5′-ends and poly-adenosine tail to its 3′-ends. Then with the assistance of spliceosomes, pre-mRNA undergoes splicing at canonical splice sites to remove introns and join exons, to form a mature and translatable linear RNA transcript with 5′ to 3′ polarity [12].

For the biogenesis of circRNA, two processes are involved: (1) a canonical alternative splicing event that joins two non-sequential exons together to form a linear RNA with skipped middle exons, and (2) the covalent circularization of the skipped middle exons by back-splicing through the ligation of a downstream splice donor site reversely with an upstream splice acceptor site, resulting in a covalently closed circRNA transcript [6]. According to the order of splicing events and different intermediates, two models of circRNA biogenesis were proposed: the lariat model and the direct back-splicing model [13].

The lariat model (or exon skipping model) starts with canonical splicing for a linear RNA with skipped exons and a long intron lariat containing these skipped exons, which is then further processed by another canonical splicing to remove the introns between them, and then back-spliced to form a circRNA. Conversely, in the direct back-splicing model, the process starts with back-splicing for a circRNA together with an exon–intron(s)-exon intermediate, which can be further processed to produce a linear RNA with skipped exons or to be potentially degraded. It is important to note that these models are not mutually exclusive and detailed lines of biochemical evidence are still required to evaluate these models. Moreover, it is still unclear under which conditions the spliceosomal machinery chooses either canonical splicing or back-splicing to generate RNA [13].

circRNAs are primarily formed by covalent joining of exons (exonic circRNAs), and these are the most studied type. However, less common types of circRNAs are formed from one or more exons that retain their flanking introns (exon–intron circRNAs) or formed only from intron sequences (circular intron RNAs) [14,15].

Exonic circRNAs are generally localized in the cytoplasm, while exon–intron and intron circRNAs are retained in the nucleus [16,17,18]. Furthermore, circRNAs can be delivered by extracellular vesicles (EVs). It has been reported that exosomes contain abundant circRNAs compared to parental cells and can transfer their specific biological activity to target cells. However, the packaging and delivery of them are yet to be identified [19]. Recently, mitochondria-located circRNAs were identified. Based on their genomic origin, they may be encoded by mitochondrial genome or nuclear genome. Less is known about their biological role, but their expression is correlated with mitochondrial function [20,21].

### 2.2. Regulation of circRNA Synthesis

Most circRNAs are less abundant than their linear mRNA counterparts [22]. Inhibiting the pre-mRNA processing machinery shifts the output of genes to circRNAs, which implies that there is a competition between circRNAs and their linear counterparts [23]. It has been reported that there is an inverse correlation between the number of linear and circular RNA molecules [12]. The selection of RNA pairing across flanking introns or within a single individual intron leads to the competition between back-splicing for circRNAs and canonical splicing for linear RNAs resulting in different expression patterns of circRNAs and linear RNAs [22]. It is possible that back-splicing is unfavorable for spliceosome assembly and thus less efficiently catalyzed by the spliceosomal machinery [13]. To overcome this natural disadvantage, Cis-elements (intronic complementary sequences, ICSs) and trans-factors (RNA binding proteins, RBPs) can promote back-splicing and circRNA production by bringing the downstream splice donor and upstream acceptor sites close together [12].

Under regulatory control, inverted repeats of *Alu* elements in flanking introns can promote exon circularization and could dramatically enhance the production of circRNAs [22]. Longer exons than average, surrounded by small introns having reversed *Alu* repeats, appear to be the main features present in the RNA circularization mechanism [16,24]. Additionally, a group of RBPs can modulate circRNA biogenesis by binding to flanking introns leading to directly drawing introns into proximity and facilitate circularization such as muscleblind (MBNL1) [12,25]. On the other hand, adenosine deaminase 1 acting on RNA (ADAR1) has been reported to suppress circRNA biogenesis [26].

Interestingly, multiple circRNAs can be identified from a single precursor RNA (alternative circularization); accordingly, circRNAs may become more abundant than their linear counterparts [24,27]. There are several proposed mechanisms for the alternative circularization process that warrant further investigation [13].

The way in which circRNAs maintain a dynamic balance is understudied. Due to their circular shape, circRNAs are closed loops lacking the free ends and hence, they are more resistant to degradation than linear RNAs with approximately 48 h cellular half-life compared to about 10 h half-life of the linear RNAs [28]. Recent studies suggested some mechanisms of circRNA degradation that act as a regulatory feedback. circRNAs destruction requires specialized endoribonucleases such as RNase L that can cleave the RNA within its loop, converting the stable loop into a linear fragment that rapidly degraded by common exonucleases [29]. Additional pathways involve YTHDF2-mediated recognition of N^6^-methyladenosine (m^6^A)-containing circRNAs [30] and UPF1/G3BP1-facilitated unwinding and subsequent degradation process of circRNAs [31].

## 3. circRNAs: Unraveling Functional Diversity and Molecular Mechanisms

**Figure 1:**
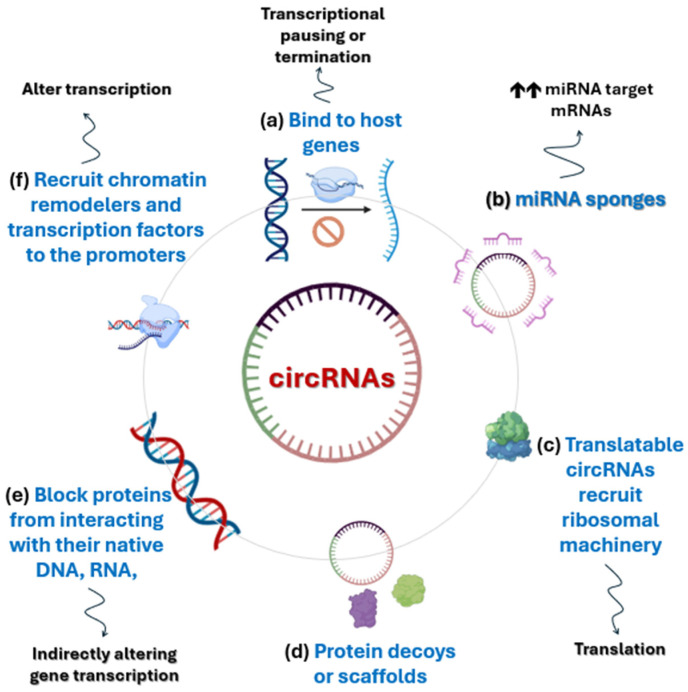
An overview of the major known biological roles of circular RNA.

Circular RNAs are recognized as critical regulators governing numerous essential biological processes under physiological conditions, including tissue development, cellular proliferation, autophagy, innate immunity, and neuronal function [32,33]. Furthermore, extensive research has illuminated the molecules’ profound involvement in disease pathology, revealing crucial biological roles across a spectrum of human illnesses, such as cancer [34,35,36], cardiovascular disease [37], neurological disorders [38], and autoimmune diseases [39]. Despite their demonstrated importance, significant knowledge gaps persist regarding the factors driving the abnormal expression of circRNAs and, more critically, the precise mechanisms through which they execute their diverse physiological and pathological functions. According to Zhou et al. [7], the known molecular actions of circRNA are broadly categorized into four main mechanisms involving interactions with other cellular components: regulation of transcription, microRNA (miRNA) sponging, serving as templates for translation into proteins, and circRNA–protein interactions. Figure 1 summarizes the major known biological roles of circRNAs across many lines of research.

### 3.1. Transcriptional Regulation and R-Loop Formation

Circular RNAs exert a foundational influence by directly modulating gene expression at the earliest stage: transcription. This regulatory capacity stems from their ability to bind to the host gene locus from which they were synthesized. Upon binding, circRNAs facilitate the formation of an RNA-DNA hybrid structure, known as an R-loop [14,17]. The presence of this R-loop can induce transcriptional pausing or premature termination, ultimately leading to the generation of exon-skipped or truncated transcripts [40,41]. This mechanism highlights circRNAs not just as downstream players, but as intrinsic regulators capable of actively shaping the primary transcriptional output of the genome.

### 3.2. MicroRNA Sponges

The most extensively documented and pivotal role of circRNAs is their function as miRNA sponges, positioning them as crucial players in the Competing Endogenous RNA (ceRNA) network. In this context, circRNAs bind to miRNAs, effectively sequestering them and preventing them from interacting with their natural mRNA targets [42,43]. The binding capacity varies greatly, with a single circRNA capable of binding multiple types and quantities of miRNAs [18]. By negatively regulating miRNA activity, through this competitive binding, circRNAs indirectly upregulate the stability and translation of target mRNAs, thereby profoundly impacting various pathological processes across human diseases [44,45].

### 3.3. Translation into Novel Peptides

circRNAs have traditionally been viewed as ncRNAs with regulatory roles. However, a significant paradigm shift occurred with the discovery of translatable circRNAs [46,47]. Unlike linear mRNAs, circRNAs lack the requisite 5′-cap and 3′-poly(A) tail for canonical eukaryotic translation initiation [48]. They overcome this hurdle by initiating translation via cap-independent manners, most commonly by possessing an Internal Ribosome Entry Site (IRES) that directly recruits the ribosomal machinery [49,50]. The resulting peptides are often truncated but possess diverse functions: while some mimic their full-length host protein counterparts, others exert functions that are entirely independent of or even antagonistic to the host gene product [51,52]. This finding dramatically expands the known range of the human proteome and introduces novel functional molecules.

### 3.4. The Power of circRNA–Protein Interactions

The function of circRNAs is heavily reliant on their ability to engage in direct interactions with proteins, making this a crucial secondary role to miRNA sponging [53,54]. The most common binding partners are RBPs, a broad class of molecules essential for regulating RNA life cycles, including maturation, transport, and translation. circRNAs leverage these interactions by adopting the role of versatile molecular platforms. They can serve as scaffolds, physically bringing together two or more proteins to assemble functional ribonucleoprotein complexes and thereby enhancing signaling efficiency. Alternatively, they can act as decoys, sequestering proteins away from their native targets and effectively inhibiting that protein’s original function [55]. This ability to manipulate protein availability and complex formation makes circRNAs indispensable orchestrators of cellular signaling pathways.

The scope of circRNA–protein interaction extends beyond simple binding, profoundly influencing existing protein–protein interactions. A single circRNA molecule can bind two or more proteins, facilitating the formation of a ternary or multi-component complex [56]. This complex formation can either stabilize and strengthen the interaction between the two proteins [57] or, conversely, act to disrupt and compromise their original association [58]. While the underlying mechanisms remain an active area of investigation, a prevailing hypothesis suggests that circRNAs may subtly alter the spatial distance between the proteins or induce an allosteric conformational change that optimizes or inhibits their fit for interaction [7]. Further advanced research and novel technologies are essential to definitively validate these intriguing structural hypotheses.

The role of circRNA as a protein binder also translates into direct functional interference by blocking proteins from interacting with their native DNA, RNA, or protein targets. For instance, circRNAs can effectively sequester DNA binding proteins, such as transcription factors, thereby negatively altering gene transcription [59,60]. In another scenario, they can bind to RBPs that are responsible for essential post-transcriptional processes like mRNA splicing, stability, and translation [61,62]. By capturing these key regulatory proteins, circRNAs indirectly intervene in the fate of mRNAs, showcasing their power to regulate gene expression outside of the nuclear transcriptional environment.

Intriguingly, circRNAs also possess the sophisticated ability to serve as nuclear recruiters, enabling them to participate directly in epigenetic regulation. Studies have confirmed that circRNAs can actively recruit essential nuclear machinery, including specific transcription factors [63,64], chromatin remodelers [65,66], and various DNA or histone modifying enzymes (e.g., those responsible for methylation and acetylation) to targeted gene promoters. By directing these complexes, circRNAs can strategically alter chromatin accessibility and switch target genes “on or off,” demonstrating their potent and widespread influence across both the transcriptional and epigenetic landscapes of the cell [67,68].

Further solidifying their role as master regulators, circRNAs are essential facilitators in the formation of circRNA–protein–mRNA ternary complexes. Within this framework, the circRNA molecule actively mediates and assists the binding of RBPs to target mRNAs. The formation of this integrated complex serves two primary purposes: it can stabilize the mRNA, thereby indirectly promoting its translation, or it can directly regulate the efficiency of translation itself, either enhancing or inhibiting the protein synthesis process [69,70]. This mechanism underscores circRNAs’ ability to operate across the entire regulatory spectrum, confirming that these molecules exert pivotal biological roles at not only the transcriptional/epigenetic levels but also through a precise and dynamic control over translation and post-transcriptional stability.

## 4. Clinical Horizons of circRNAs

### 4.1. Overview of the Methodological Landscape for circRNA Studies

The accurate study of circRNAs hinges upon specialized detection and quantification techniques [71]. While Northern blotting historically served as an initial validation assay, its inherent complexity and low sensitivity have led to its limited use [72]. Today, the gold standard for confirmation and initial quantification relies on real-time polymerase chain reaction (RT-PCR) or quantitative PCR (qPCR), which utilizes divergent primers engineered to specifically amplify the unique back-splicing junctions characteristic of circular transcripts. For achieving the highest levels of precision and absolute quantification, digital droplet PCR (ddPCR) is increasingly employed to accurately determine circRNA copy numbers [18]. Furthermore, to map the location of these molecules within the cell, fluorescence in situ hybridization (FISH) uses targeted fluorescent probes to visualize the crucial subcellular localization of specific circRNAs [73,74]. Parallel to these wet-lab methods, sophisticated bioinformatics pipelines (e.g., circRNA_finder, find_circ, CIRCexplorer) analyze RNA-sequencing data by identifying reads that span the back-splicing junctions, enabling accurate discrimination between circular and linear transcripts and fueling subsequent functional analysis [75,76].

To truly decipher the regulatory roles of circRNAs, specialized assays are required to map their interactions with proteins. The foundational technique for identifying circRNA–protein partnerships is the circRNA pull-down assay [77]. This method uses a biotin-labeled probe, complementary to the circRNA of interest, to isolate the molecule from cell lysates. The co-purified proteins are then rigorously identified using mass spectrometry or confirmed via Western blotting [74]. Conversely, when the regulatory protein is the target, RNA immunoprecipitation (RIP) is employed: an antibody pulls down the suspected RNA-binding protein (RBP), and the associated circRNA is subsequently detected and confirmed via PCR or sequencing [78]. For high-resolution mapping of the exact interaction sites, crosslinking-based immunoprecipitation (CLIP) variants (such as HITS-CLIP or eCLIP) use UV light to create covalent bonds between the circRNA and the RBP, yielding site-specific data [79]. Finally, the electrophoretic mobility shift assay (EMSA) provides a crucial in vitro validation step, confirming the direct physical formation of the circRNA–protein complex [80]. Collectively, these techniques are indispensable for defining the composition and specificity of the circRNA-centered regulatory networks.

### 4.2. Emerging Roles as Diagnostic Biomarkers and Therapeutic Agents

The intrinsic molecular characteristics of circRNAs present them as ideal candidates for use as future diagnostic biomarkers. Their defining feature, a stable closed-loop structure, imparts a significantly longer half-life and enhanced resistance to RNase degradation compared to linear RNAs [81]. This stability ensures that they remain reliably detectable in biofluids. Indeed, over 2400 circRNAs have been detected in human whole blood, with total circulating levels rivaling those found in the brain, underscoring their high abundance and accessibility [82]. Consequently, researchers are actively harnessing the diagnostic potential of circRNAs across various diseases [83]. Their applications extend beyond early-stage disease detection, also proving valuable in predicting prognosis and monitoring disease progression [84], making them powerful non-invasive tools for clinical assessment.

Beyond diagnostics, the therapeutic promise of circRNAs is rapidly gaining traction, driven by their tissue- and cell type-specific expression patterns across diseases such as cancer, cardiovascular disorders, and nervous system diseases [83]. Following the success of modern RNA therapies, circRNAs are being aggressively explored as a next-generation replacement for traditional linear mRNAs. Their superior stability due to exonuclease resistance, coupled with the lack of free ends, enables endogenous circRNAs to potentially bypass the typical RNA-mediated innate immune response that challenges other RNA agents [85]. Despite ongoing discussions regarding their immunogenicity, circRNAs remain one of the most compelling candidates for future RNA-based treatments. Achieving safe and effective delivery to target organs is paramount, and current research employs a range of sophisticated technologies, including adeno-associated viruses (AAVs), lipid nanoparticles, and plasmids, to overcome delivery problems [86,87].

## 5. circRNAs: Emerging Players in Host–Parasite Interactions

**Figure 2:**
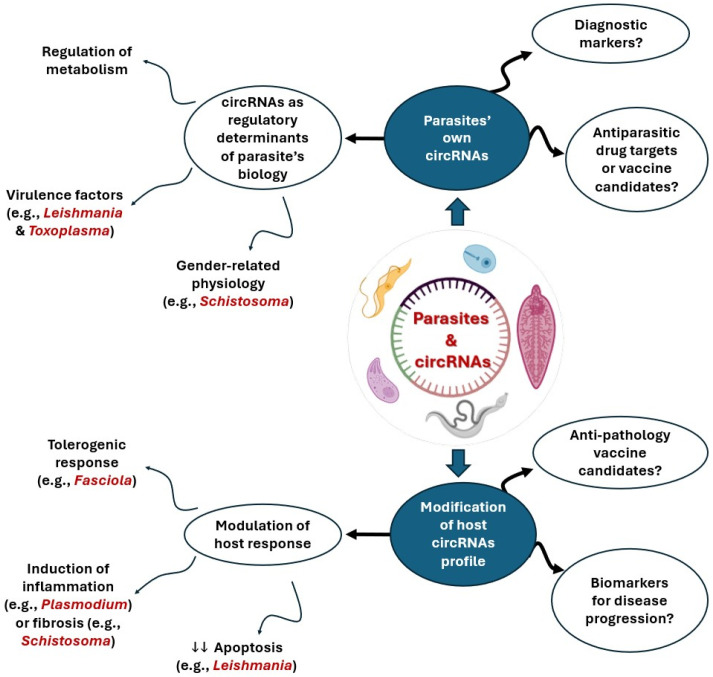
Schematic presentation of the available knowledge of circRNAs and parasites across many lines of research. (?) denotes potential applications of circRNAs.

circRNAs emerge as potential players in host–parasite interactions. They have been investigated in a variety of parasitic infections but the overall picture is still missing in many different scenarios of host–parasite interrelationships. Herein, we provide an overview of circRNAs in parasites, focusing solely on major human parasites of public health significance. Figure 2 summarizes the available knowledge of circRNAs in parasites across many lines of research.

### 5.1. Plasmodium Species

Malaria is a widespread devastating parasitic infection caused by multiple-stage Apicomplexan parasites of the genus *Plasmodium*. Four main species were long recognized: *Plasmodium falciparum* (*P*. *falciparum*), *P. vivax*, *P. ovale*, and *P. malariae*. An additional species, *P. knowlesi*, has been recently identified as the fifth human malaria parasite. Human transmission occurs via the bite of female *Anopheles* mosquitoes [88]. Nearly half of the world population is at risk of malaria [89]. In 2024, there were an estimated 282 million malaria cases and 610,000 deaths—roughly 9 million more cases than in 2023. Africa continues to bear the heaviest toll, with 11 countries accounting for about two thirds of clinical cases and deaths. Efforts at reducing the malaria mortality rate regrettably remains far off track [90].

*Plasmodium falciparum* is the deadliest species, accounting for most morbidity and almost all fatalities of malaria. It is the most common species in Sub-Saharan Africa. The parasite is associated with severe forms of malaria, and is notorious for its tendency to develop resistance to antimalarial chemotherapy [89]. Unsurprisingly, most malaria research focuses on *P. falciparum* to better understand host–parasite dynamics, to develop reliable diagnostic tools, and to design novel antimalarial agents or, the eagerly desired, effective vaccines. Several nonhuman malaria models have been used to study various aspects of *Plasmodium* infection. Among these, rodent and nonhuman primate malaria models are the most optimal [88].

The genomes of several *Plasmodium* species have now been sequenced and nearly half of the identified genes are of unrecognized functions [91]. Recent research has shown that *Plasmodium* species require tight transcriptional and post-transcriptional control in order to navigate its complex transmission events in the host and the vector. The exact mechanisms of gene expression control are still largely unknown and much research is needed in this domain [10]. Several species of ncRNAs have been identified in *Plasmodium* spp., particularly in *P. falciparum*. Lodde et al. [92] reviewed the role of ncRNAs in malaria, including miRNAs, lncRNAs, and circRNAs with special emphasis on the possible role of these ncRNAs as biomarkers for *Plasmodium* infections. Across several studies, many miRNAs and lncRNAs were found to modulate host gene expression and mediate features like antigenic variation and parasite virulence as well as life cycle changes [10,93,94,95].

In addition to miRNAs and lncRNAs, Wei et al. [96] reported the presence of intermediate-size ncRNAs (is-ncRNAs) in intra-erythrocytic *P. falciparum*, strain 3D7. The expression levels of the antisense RNAs correlated with those of their cis-encoded sense RNA counterparts, implying that these is-ncRNAs are involved in the regulation of gene expression of the parasite. Surprisingly, very few reports are found in the literature as regards circRNAs in *Plasmodium*. In fact and generally speaking, the research on ncRNAs in malaria is emerging and indeed more attention is needed to explore the role of circRNAs in malaria.

Unlike metazoans, protozoan organisms like *P. falciparum* have very short introns (~100 nucleotides or shorter), yet they can produce circRNAs. Wang et al. [9] were the first to show that *P. falciparum* expresses low abundance circRNAs. They elaborated that few genes of *P. falciparum* show documented examples of canonical alternative splicing, challenging the view that all circRNAs are by-products of alternative splicing or piggyback on signals used in alternative RNA processing. As the authors concluded, these findings denote that circRNA may be an ancient, conserved feature of gene expression programs in eukaryotes.

Later, Broadbent et al. [10] were able, via strand-specific RNA sequencing in *P. falciparum*, to unveil developmentally regulated ncRNAs, including circRNAs and lncRNAs. Owing to deep sequencing, they predicted hundreds of *P. falciparum* circRNAs, six of which were validated experimentally. Short circRNAs substantially outnumbered longer circRNAs. Of equal importance is the finding that the experimentally validated circRNA candidates each contained predicted human miRNA binding site. The latter opens the door for many speculations about the role of circRNAs in *Plasmodium*–host interactions. Interestingly, this study showed that a highly expressed, five-exon antisense RNA seems to regulate *P. falciparum* gametocyte development, *PFGDV1*, a gene required for early sexual commitment events.

In a recent study of a rodent malaria model (*P. yoelii* 17XL in BALB/c mice), Xu et al. [97] explored the role of circRNAs during intraerythrocytic *Plasmodium* infection. Using deep sequencing, they found that *Plasmodium* infection substantially reshapes the host circRNA landscape. Out of ~1200 circRNAs shared between infected and uninfected mice, 60 were upregulated and 71 were downregulated after infection. Remarkably, all modified circRNAs were host-derived, denoting that *Plasmodium* is manipulating host RNA regulation rather than contributing its own circRNAs. From these, the authors narrowed down 11 differentially expressed circRNAs based on predicted function—either the ability to sponge miRNAs or the potential to encode proteins via IRES. This is noteworthy given that circRNAs are usually thought of as noncoding, but several are predicted to be translatable in this study.

Further, in the previous study and through functional enrichment analyses [Gene Ontology (GO) and Kyoto Encyclopedia of Genes and Genomes (KEGG)], it has been showed that the target genes of these circRNAs cluster strongly in inflammatory and immune signaling pathways, especially mitogen-activated protein kinase (MAPK) signaling, NF-κB signaling, and TGF-β and chemokine pathways. These findings were confirmed by measurement of cytokines and proteins involved in inflammation. Collectively, *Plasmodium* seems to indirectly trigger an inflammatory response by rewiring host circRNA expression, which in turn acts as miRNA sponges, altering post-transcriptional regulation, and/or encodes small functional proteins that participate in inflammatory signaling. Interestingly, the study also identified an intriguing set of novel circRNAs that appeared only after infection. These do not originate from the parasite genome, thus the authors suggested that *Plasmodium* may influence host back-splicing mechanisms or m^6^A RNA modifications, driving the production of novel circRNAs under inflammatory stress.

Overall, despite the heavy impact of malaria on human health and the inherent difficulties in its diagnosis and treatment, our knowledge about the role of ncRNAs, particularly the circRNA subset, in malaria is quite limited and sketchy. Systematic well-designed studies, perhaps through conjoint efforts of many labs, are needed to characterize different circRNAs and elucidate their role during *Plasmodium* infections. If successful, these efforts should provide insights about host–*Plasmodium* dynamics, hopefully culminating into development of better diagnostics or discovery of drug targets or vaccine candidates.

### 5.2. Toxoplasma gondii

Toxoplasmosis is a widespread infection caused by the Apicomplexan protozoan *Toxoplasma gondii* (*T. gondii*), a parasite estimated to infect nearly one-third of the global human population [98]. While the infection typically remains asymptomatic in healthy individuals, it poses a severe threat to those with compromised immune systems, such as patients with AIDS, where it can manifest as debilitating neurological disorders. Once the initial infection occurs, the parasite establishes a permanent, latent presence within the host, which carries the risk of reactivation if the host’s immune defenses weaken [99]. Current medical interventions for toxoplasmosis are hindered by the limitations of existing drugs and the absence of effective human vaccines [100]. Consequently, there is an urgent scientific mandate to better understand the complex biological dialogue between *T. gondii* and its host. Specifically, researchers are focused on decoding the molecular mechanisms that drive the parasite’s pathogenesis during its lytic cycle to identify potential targets for future treatments.

Recent advancements in biotechnology have allowed researchers to examine how *T. gondii* manipulates its environment through global transcriptomics [101,102,103] and proteomics [104,105]. These studies have demonstrated that the parasite significantly alters host gene expression and protein levels across various tissues. Furthermore, infection has been shown to disturb the expression of ncRNAs both in living organisms and in laboratory cultures [106,107,108]. The parasite effectively “hijacks” the host’s cellular machinery, modulating essential immune pathways such as cytokine production and programmed cell death, or apoptosis [109].

Research has consistently demonstrated that the presence of *T. gondii* leads to significant shifts in the host’s circRNA landscape. A primary example is the work of Wang et al. [110], who utilized RNA sequencing to examine human foreskin fibroblasts (HFFs) maintained in Dulbecco’s modified Eagle’s medium throughout the parasite’s lytic cycle. Their investigation uncovered a massive range of differentially expressed circRNAs, numbering from several hundred to thousands, depending on the specific time points following the initial infection. Through the application of GO and KEGG enrichment analyses, the researchers determined that these host-derived circRNAs are intricately connected to metabolic processes, signal transduction, and pathways governing immunity and apoptosis. These findings strongly imply that circRNAs play a functional role in regulating cellular behavior and immune defenses during the progression of toxoplasmosis.

In addition to cellular studies, animal models have provided insight into organ-specific responses, particularly within the liver. For example, Zou et al. [111] conducted a profile of liver circRNAs in mice during both the acute and the chronic phase of *T. gondii* exposure. Their data identified 265 differentially expressed circRNAs during the acute stage and 97 during the chronic stage when compared to healthy controls. These specific RNAs were notably enriched for GO terms associated with immune functions, such as the regulation of T cell activation, immune receptor activity, and the positive regulation of cytokine production. A specific example highlighted in their study was a differentially expressed circRNAs that integrated into a network with immune-regulatory miRNAs, specifically mmu-miR-146a-5p and mmu-miR-150-5p, suggesting that the parasite triggers a broad hepatic circRNA response to modulate inflammatory and immune outcomes.

The impact of *T. gondii* extends to the central nervous system, where genome-wide profiling has revealed distinct circRNA involvement in mouse brains. Zhou et al. [112] observed that acute infection dysregulated 76 host circRNAs, the majority of which were downregulated, whereas chronic infection resulted in the alteration of only 3 circRNAs. Gene Ontology analysis indicated that the host genes responsible for producing these acute-phase circRNAs were primarily involved in “ion binding” and “response to stimulus” functions. By constructing complex circRNA–miRNA–mRNA networks, the authors proposed a mechanism where host circRNAs act as “sponges” for miRNAs. This interaction potentially interferes with or directs neuronal and immune signaling pathways within the infected brain tissue, further illustrating the parasite’s influence on host molecular architecture.

Although specific diagnostic tests have not yet been developed, these studies validate that circRNAs are unusually stable RNA species. In other contexts (e.g., cancer, liver disease), circRNAs have been proposed as stable prognostic biomarkers. By analogy, infection-specific circRNA signatures (in blood or tissue) could be evaluated as biomarkers of toxoplasmosis, pending further validation. In summary, recent work indicates that host circRNAs are dynamically regulated during *T. gondii* infection and likely contribute to host–parasite interactions via immune and metabolic pathways.

### 5.3. Leishmania Species

Leishmaniasis is a major neglected tropical disease caused by *Leishmania* parasites [113]. It represents the third most burdensome parasitic infection worldwide [114], with more than 20 pathogenic species across 98 countries [115,116]. The disease manifests typically in 3 major forms: cutaneous, mucocutaneous, and visceral leishmaniasis [117]. Diagnosis traditionally relies on parasite detection in tissue aspirates [118], a method that is invasive, often painful, and prone to false negatives [119]. The nonspecific clinical profile of visceral leishmaniasis further complicates the diagnosis [120,121], underscoring the need for novel, less invasive diagnostic tools.

Importantly, macrophages serve as the primary host cells for *Leishmania*, and infections cause marked alterations in macrophage metabolic and transcriptional programs [122,123]. Recent transcriptomic investigations have highlighted substantial shifts in ncRNA landscapes during infection. Li et al. [124] profiled serum RNA signatures in leishmaniasis patients and identified extensive dysregulation of 4664 circRNAs and 57 miRNAs. The host genes corresponding to these circRNAs were enriched in pathways such as G2/M cell cycle progression and ubiquitin-mediated proteolysis, and a circRNA–miRNA–mRNA network was proposed, suggesting multilayered post-transcriptional regulation during disease progression.

Based on these findings, Alizadeh et al. [125] profiled circRNA expression in an in vitro model using cultured macrophages infected with *Leishmania infantum* (*L. infantum*) and *Leishmania tropica* (*L. tropica*). Total RNA was extracted from both the host cells and their culture supernatants, and the expression levels of 30 selected circRNAs were quantified using reverse transcription quantitative polymerase chain reaction (RT-qPCR), with functional roles predicted via bioinformatics platforms such as CircNET and CircAtlas 3.0. Five circRNAs, hsa-circ-0032822, hsa-circ-0008603, hsa-circ-0008042, hsa-circ-0005320, hsa-circ-0000869, were differentially expressed, with hsa-circ-0032822 showing the strongest upregulation and a four-fold higher concentration in culture supernatants. Its previously reported role in promoting S-phase arrest and reducing apoptosis [126] aligns with observations suggesting that its overexpression may extend macrophage lifespan and thereby support parasite persistence [125]. Furthermore, its detection across all experimental groups allowed the authors to suggest that hsa-circ-0032822 may represent a common host factor involved in the cellular response to infections by both *L. infantum* and *L. tropica*, warranting further dedicated functional research to elucidate its precise mechanism in parasite survival within the macrophage.

Species-specific signatures were also evident. Alizadeh et al. [125] reported that hsa-circ-0008042, was significantly elevated only in *L. tropica*-infected macrophages, highlighting its potential as a species-specific diagnostic marker. Similarly, hsa_circ_0008603 was upregulated in *L. infantum* but downregulated in *L. tropica*. Interestingly, such circRNA is encoded by the *ALDH3A2* gene, a member of aldehyde dehydrogenase (ALDH) family. ALDH enzymes are known to be vital for the parasite’s defense against host-generated oxidative stress and are directly implicated in *Leishmania* virulence [117,127,128,129]. Hence, its increased expression may contribute to enhanced survival of *L. infantum* within macrophages. Finally, the detection of hsa-circ-0032822, hsa-circ-0008603, and hsa-circ0000869 in supernatants [130] supports the concept of circulating circRNAs as minimally invasive biomarkers, offering a promising alternative to bone marrow aspiration in disease monitoring [119].

### 5.4. Trypanosoma Species

*Trypanosoma brucei* (*T. brucei*) is an insect-borne parasitic protist of the Kinetoplastea class. It is widely recognized as the cause of African trypanosomiasis, also known as sleeping sickness in humans. The disease poses a threat to millions and imposes substantial economic costs throughout Sub-Saharan Africa [131]. *T. brucei* features a unique mitochondrial nucleoprotein assembly known as the kinetoplast, with around 20 genes (2 rRNAs and 18 mRNAs) that primarily code for proteins of the mitochondrial electron transport chain [132]. The mitochondrial mRNAs transcribed from this kDNA undergo extensive processing, including modifications at their ends and addition or removal of uridine residues [133].

Crucially, Smoniewski et al. [134] reported that *T. brucei* produces mitochondrial circRNAs derived from multiple mRNA transcripts in both its insect and bloodstream life cycle stages. Using circular RT-PCR and deep sequencing, they confirmed that these circRNAs have their 5′ and 3′ ends covalently joined. These circRNAs represent a distinct subset of the total mitochondrial mRNA population that exhibit markedly shorter or fewer 3′ UTR tails compared with their linear mRNA counterparts. Only a minority of circRNA reads contained 3′ adenine-rich extensions, and when present, these tails were significantly shorter and less A-rich than those observed in the total mRNA population. The authors proposed that circularization may occur prior to polyadenylation, implying an alternative maturation or degradation route that deviates from the canonical mRNA life cycle

To date, *T. brucei* remains the only trypanosomatid in which circRNAs have been clearly described. These parasite-derived circRNAs are an emerging area of interest, and their biological roles remain unknown. Smoniewski et al. [134] suggested that these circRNAs may be included in the regulation of mitochondrial gene expression, modulation of RNA stability, or participation in specialized RNA–protein complexes. Because circRNAs lack the canonical 5′ cap and 3′ tail, they may evade exonucleolytic decay and thus follow a distinct stability trajectory compared with linear transcripts. Smoniewski et al. [134] proposed that circularization might “rescue” mRNAs from degradation or mark them for an alternative processing pathway. Further investigation is needed to determine whether trypanosome circRNAs can encode peptides, via IRES, as observed in certain viral circRNAs, or act as scaffolds for mitochondrial RNA-binding proteins.

### 5.5. Schistosoma Species

Schistosomiasis is a neglected tropical disease caused by blood flukes of the genus *Schistosoma,* prevalent in many developing countries in tropical Africa, the Middle East, Asia, and Latin America. The main species infecting humans are *Schistosoma haematobium* (*S. haematobium*), *S. mansoni*, and *S. japonicum*. About 800 million people are at risk of infection with schistosomes and over 250 million people are infected in tropical and subtropical regions, leading to an estimated ~280,000 deaths annually [135,136,137].

*Schistosomes* are the only trematodes that have evolved separate sexes and possess a unique reproductive developmental process involving male and female worm interactions and complex molecular regulatory events [138]. Previous research has demonstrated dramatic changes in gene expression in both genders associated with pairing and sexual development. The ncRNAs are abundant throughout the genome of schistosomes including miRNAs that are found to be involved in the regulation of ovarian development in *S*. *japonicum* [139,140,141,142].

circRNAs are evolutionarily conserved across species. Giri et al. [143] identified a total of 2636 circRNAs of parasite origin in adult worms of *S. japonicum*, predominantly derived from exons: 748 circRNAs in adult females and 1888 in adult males. These gender-associated circRNAs expression suggests that circRNAs may have potential regulatory roles in schistosome development, biological functions and sexual differentiation. Furthermore, they performed bioinformatic analysis of two circRNAs (circ-003048 and circ-004690) and their predicted binding sites of miRNA (sja-miR-1, sjamiR-3504 and sja-miR-133). Previous reports indicated that these miRNAs show differential expression between male and female worms, and gender-specific functions related to growth, reproductive development and egg production [140,144,145].

Adult female *Schistosoma* produces hundreds of eggs, which are critical in the pathology and clinical symptoms of schistosomiasis. Eggs of *S. mansoni* and *S. japonicum* are deposited in intestinal wall and the liver and initiate a distinct immune response leading to granuloma formation. This inflammatory environment leads to the activation of hepatic stellate cells (HSCs), the primary source of excessive collagen deposition, which ultimately leads to liver fibrosis [146,147,148].

The role of circRNAs in the development of liver fibrosis in *S. japonicum* infection was investigated by Dai et al. [148]. Second-generation high-throughput sequencing of circRNAs in HSCs, isolated from livers of mice infected with *S. japonicum* at 6, 9 and 12 weeks post infection, identified 489 differentially expressed circRNAs: 194 circRNAs were up-regulated and 295 were down-regulated. Among them, circGsr-0002 was highly expressed in both activated HSCs of *S. japonicum*-infected mice. Moreover, they showed that circGsr-0002 exerted a miRNA sponge function of miR-383-3p which is responsible for inhibition of HSCs activation. Furthermore, for functional validation of circGsr-0002, small interfering RNA of circGsr-0002 was used to knockdown circGsr-0002. Interestingly, mRNA and protein levels of alpha smooth muscle actin and collagen type I alpha 1 were significantly downregulated upon knockdown of circGsr-0002 with inhibition of HSCs activation, subsequently attenuated the liver fibrosis. Therefore, circGsr-0002 is significantly involved in schistosome-induced liver fibrosis and it represents a potential marker for “grading” the severity of liver damage.

### 5.6. Fasciola Species (Liver Flukes)

Fascioliasis is a major parasitic infection triggered by the liver flukes *Fasciola gigantica* (*F. gigantica*) and *F. hepatica*, resulting in substantial financial burdens for the livestock industry and posing a grave risk to human health [149]. On a global scale, the damage to livestock production alone is estimated to surpass $3 billion annually [150]. Despite these staggering consequences, our grasp of the specific host defense mechanisms activated against *Fasciola* remains restricted. Consequently, investigating the molecular dialogues between these flukes and their hosts is essential for deciphering the host immune response and pinpointing specific molecular targets that could lead to the development of effective new therapies.

Recent research by Wang et al. [151] explored how the host molecular environment reacts to the parasite by exposing goat peripheral blood mononuclear cells (PBMCs) to the excretory–secretory products (ESPs) of *F. gigantica* in a laboratory setting. This study revealed a significant shift in the host’s circRNA landscape, identifying 136 differentially expressed circRNAs, specifically 83 that were upregulated and 53 that were downregulated, when compared to control groups. These findings, which were confirmed using qRT-PCR validation, demonstrated that *Fasciola* ESPs have the direct capacity to modify host circRNA expression. Furthermore, GO and KEGG analyses indicated that these changes affect genes tied to host metabolism, receptor signaling, disease progression, and the overall immune response, providing a solid foundation for future research into how ncRNAs mediate the immune-modulating effects of the parasite.

A key finding from the research by Wang et al. [151] is that KEGG pathway analysis highlighted the involvement of the B-cell receptor signaling and the tumor necrosis factor (TNF) signaling pathways, both of which are fundamental to the processes of inflammation and immune activation. This suggests that the alterations in circRNA induced by the parasite intersect with specific pathways, such as TNF and immune receptors, which the liver fluke likely manipulates to bypass or suppress the host’s natural immune defenses. While the previous study focused on circRNAs, other *Fasciola* research shows that parasite miRNAs can enter host macrophages and dampen pro-inflammatory responses. Together, these findings suggest that *Fasciola* modulate host non-coding RNA networks via its ESPs. The observed circRNA shifts in host PBMCs imply that circRNAs may participate in the “tolerogenic” immune environment favored by the fluke. For example, the induction of circRNAs regulating metabolic genes could support the tissue repair response [151].

### 5.7. Echinococcus Species

Cystic echinococcosis (CE) is a significant zoonotic parasitic infection caused by the *Echinococcus granulosus* (*E*. *granulosus*) tapeworm. The biological progression of this parasite relies on a complex transmission cycle involving two distinct hosts: dogs serve as the definitive hosts, while various livestock, particularly sheep, function as the primary intermediate hosts. Humans typically enter this cycle as accidental intermediate hosts. While the parasite is capable of infecting nearly any tissue or organ in the human body, clinical data indicates a high preference for the liver and lungs, which account for approximately 70% and 20% of reported cases, respectively [152]. Within the tissues, the parasite’s larval stage, known as the metacestode, matures into fluid-filled structures called hydatid cysts. These cysts possess a sophisticated anatomical hierarchy consisting of an inner germinal layer, a middle laminated membrane, and an outer fibrous membrane. The interior is filled with hydatid fluid (HF) secreted by the germinal layer. This fluid is essential for the parasite’s survival, as it provides vital nutrients and houses the invaginated tissue layers known as protoscoleces (PSCs) [153].

The interaction between the host’s immune system and the metacestode is a critical factor in determining the growth rate and overall characteristics of the cyst [154,155]. However, the pericystic wall presents a formidable obstacle to medical intervention, acting as a physical barrier that prevents effective drug penetration. Due to this limited pharmacological efficacy against the pericyst, surgical removal remains the primary treatment modality for hydatid disease. This has prompted researchers to investigate the regulatory mechanisms governing the host–parasite relationship, specifically focusing on how small RNAs fine-tune gene expression at the infection site [156,157].

Research has demonstrated that a continuous exchange of biological macromolecules occurs across the laminated layer via constant vesicular trafficking [158]. The parasite produces exosome-like vesicles (ELVs) that facilitate this communication. It is hypothesized that extracellular inositol hexaphosphoric acid binds to proteins on these ELVs, serving as a “dynamic anchorage” that enables them to traverse the laminated layer [159]. These parasite-derived ELVs are instrumental in intercellular signaling and pathology, carrying various ncRNAs that modulate the host’s immune response [160,161].

Zhang et al. [156] isolated and characterized the ELVs produced from in vitro cultures of *E. granulosus* PSCs and HF obtained from naturally infected sheep. The isolated PSC-ELVs and HF-ELVs were subjected to high-throughput sequencing, revealing many miRNAs, lncRNAs, and circRNAs in PSC-ELVs and HF-ELVs. Importantly, Zhang et al. [156] reported that PSC-ELVs carried higher numbers and levels of circRNAs than HF-ELVs. Furthermore, bioinformatic analysis constructed parasite circRNA–miRNA–mRNA networks, suggesting that *E. granulosus* circRNAs might regulate parasite and host genes at the infection interface. These findings imply that *E. granulosus* secretes circRNAs that could influence host–pathogen communication.

The impact of CE on the host is further evidenced by significant changes in the expression of host circRNAs within infected tissues. Microarray analysis of pericystic tissue from CE patients revealed 343 significantly altered circRNAs, with 177 being upregulated and 166 downregulated compared to healthy liver tissue [157]. Specifically, four circRNAs, hsa_circRNA_006773, 049637, 104349, and 406281, were validated as being differentially expressed. These molecules are believed to act as “sponges” for host miRNAs, thereby influencing pathways related to organic cyclic compounds and potentially serving as future biomarkers or therapeutic targets.

Beyond CE, alveolar echinococcosis (AE), caused by *E. multilocularis*, presents a more aggressive form of hydatid disease with a poorer prognosis [162,163]. Studies involving AE patients have identified 59 distinct circRNAs in serum exosomes that differ from healthy controls, providing a basis for noninvasive diagnostic tools [164]. While specific functional characterizations of these individual circRNAs are ongoing, the established variance in host circRNA secretion confirms that the body undergoes a systemic regulatory shift in response to the presence of the *E. multilocularis* parasite.

Experimental mouse models have provided deep insights into the cellular host response to infection. Research mapping the host circRNA response in murine liver cells, including hepatocytes, stellate cells, and Kupffer cells, identified over 6000 host circRNAs, many of which were significantly altered during the progression of the disease [165]. Furthermore, studies focusing on the early stages of infection (2–15 days) have linked thousands of circRNAs to immune pathways such as T cell activation and antigen presentation. For instance, the host circRNA mmu_circ_0008646 was predicted to bind miR-466b-3p and miR-574-5p (a miRNA known to regulate inflammation via NF-κB), suggesting a circRNA–miRNA axis modulating the early immune response in AE [166].

Overall, Table 1 and Table 2 provide a comprehensive summary of the current knowledge regarding circRNA expression and the possible functional roles across the various parasitic species discussed in this review.

## 6. Challenges and Pitfalls

The great advancement in circRNA research has disclosed much about circRNA biology and its multiple implications in various diseases including some parasitic infections. However, several challenges remain in identification and characterization of circRNAs. Current tools for isolation, detection and validation of circRNAs are of high cost, time consuming, labor-intensive and exhibit varying sensitivity rates, mostly because of the diverse biogenesis, variable length, low abundance and sequence complexity of circRNAs (Limitations of these wet-lab techniques are discussed by Chakravarthi et al. [167]). While the number of bioinformatics’ predictive models for circRNA function and disease association is growing, their reliance on incomplete datasets limits their overall accuracy [168]. Li et al. [169] designed a novel computational pipeline named COL for identifying putatively functional back-splicing and circRNAs. Furthermore, without experimental validation, the computational models prone to inaccurately attribute the origins of genetic material (host/parasite origins) and identifying technical sequencing artifacts as functional back-splice junctions [169,170]. Developing integrated methods may help to solve this issue. Moreover, the development of a gold standard set of circRNAs is a necessary step to benchmark the performance of different circRNA identification tools for consistent reporting on circRNAs [171,172].

The roles of circRNAs in parasite biology and host–parasite interactions remain poorly understood. Although many studies have investigated circRNA expression in parasitic infections, most remain descriptive and transcriptome-based. Across the parasites discussed in this review, research has primarily relied on RNA sequencing, differential expression analysis, and bioinformatic prediction to construct putative circRNA–miRNA–mRNA networks, providing valuable expression profiles. With the exception of a single study that incorporated functional validation [148], nearly all reports have not performed mechanistic experiments such as knockdown, overexpression, luciferase assays, or RNA pull-down to confirm predicted regulatory roles. Consequently, the biological relevance and causal contribution of circRNAs to parasite survival, host immune modulation, or disease progression remain largely speculative. This gap highlights the need for studies moving beyond transcriptomic profiling toward experimental validation to elucidate circRNA function and their potential as diagnostic or therapeutic targets in parasitic diseases. Moreover, circRNA profiles vary widely across parasite species and different developmental stages of the parasite. Therefore, studies on parasite circRNAs must be species-specific and stage-specific to provide accurate insights about circRNA [173,174,175].

Additionally, several technical challenges of circRNA synthesis impede its applications for diagnostic, therapeutic or vaccine purposes including suboptimal cyclization efficiency and high costs of reagents. Moreover, large-scale production necessitates standardization and optimization of manufacturing protocols and specialized equipment. Furthermore, in vitro-transcribed circRNAs exhibit pleiotropic bioactivity that carries risks of off-target interactions and unexpected immunogenic consequences. This biological complexity necessitates extensive safety evaluation of circRNAs including their immunogenic profiles [176].

## 7. Concluding Remarks

Our knowledge of ncRNAs is constantly expanding in biology and medicine, with the aim of developing better diagnostic tools for diseases and boosting our therapeutic arsenal. The domain of infectious diseases, human parasitology included, is no exception. For parasitic infections, miRNAs and lncRNAs have received much attention, whereas our knowledge of circRNAs is unfortunately lagging behind. Typically, parasites elaborate their own sets of circRNAs, which supposedly impart unique signatures to them. This could be of help for biologists involved in the tedious task of the taxonomic classification of parasites. Owing to their specificity and relative stability, circRNAs are candidates to be useful diagnostic markers. Furthermore, unveiling circRNAs with indispensable functions for the parasites could lead to the discovery of novel therapeutic targets or vaccine candidates.

Meanwhile, the parasites modify the host landscape of circRNAs in order to sustain favorable host–parasite dynamics. Some circRNAs are upregulated and others are downregulated. The identification of the characteristic pattern of host circRNA alteration during infection may help in finding diagnostic or prognostic biomarkers for the disease. Characteristically, functional analyses show that most altered circRNAs affect gene expression linked to host immune and inflammatory pathways—a fact that could pave the way for developing new therapies, via circRNA mimetics or antagonists, for parasitic diseases or even non-communicable diseases.

To conclude, circRNAs emerge as potential players in the complex interactions between the parasites and their hosts. However, the research on circRNAs in parasitic infections is still in its infancy, with a paucity of meaningful studies. As yet, there are no available circRNA-based therapies or diagnostic tools in the field of human parasitology, underscoring the pressing need for further investigation into the roles of circRNAs during parasitic diseases. As outlined before, many obstacles exist, but with coordinated, purposeful, and well-designed preclinical research, and with the aid of the rapidly developing tools in molecular biology and bioinformatics, we should be able to harness these molecules in human medicine in the near future. Generally speaking, circRNAs require a vehicle for their delivery inside the cells such as vectors or nanoparticles. Following the era of global-scale vaccination against COVID-19 using nucleic acid constructs, the use of these molecules in medical therapy might be witnessed soon.

## Figures and Tables

**Table 1:** Current research on circRNAs in protozoan parasites.

Parasite	Experimental Model & Sample	No. of Reported circRNAs	Detection Method	Functional Evidence Level	Proposed Biological Role	Ref.
*Plasmodium falciparum*	Cultured *P. falciparum* strain 3D7 clone (ATTC) in human RBCs; Parasite developmental stages	Hundreds predicted; 6 experimentally validated (Parasite-derived)	RNA-seq	Predicted human miRNA binding sites; Bioinformatic analysis	Possible host–parasite interaction role	[10]
*Plasmodium yoelii*	In vivo murine model (BALB/c); Mouse blood	60 upregulated, 71 downregulated (Host-derived)	RNA-seq + qRT-PCR	Correlation-based; no functional validation; GO/KEGG + cytokine assays	circRNAs linked to MAPK, NF-κB, TGF-β pathways; predicted miRNA sponge and possible protein-coding potential	[97]
*Toxoplasma gondii*	In vitro cell culture (lytic cycle); Human foreskin fibroblasts	Hundreds to thousands (time-dependent) (Host-derived)	RNA-seq + qRT-PCR	Correlation-based; no functional validation	Metabolic processes, signal transduction, and pathways governing immunity and apoptosis	[110]
	In vivo murine model (Acute & chronic); Mouse liver	256 (acute), 97 (chronic)	RNA-seq	Correlation-based; Bioinformatics network analysis	Regulation of T cell activation & cytokine production (predicted)	[111]
	In vivo murine model; Mouse brain	76 (acute), 3 (chronic)	RNA-seq	Correlation-based; circRNA–miRNA–mRNA network modeling	Ion binding; stimulus response; predicted miRNA sponge activity	[112]
*Leishmania* spp.	Leishmaniasis human patients; Human serum	4664 dysregulated circRNAs	RNA profiling (transcriptomic analysis)	Correlation-based; network prediction only	circRNA–miRNA–mRNA network proposed; enrichment in G2/M cell cycle and ubiquitin-mediated proteolysis pathways	[124]
*Leishmania infantum* & *L. tropica*	In vitro macrophage infection model; Cultured human macrophages + supernatants	30 selected circRNAs screened; 5 differentially expressed; Species-specific expression pattern (Host-derived)	qRT-PCR	Correlation-based; Bioinformatic functional prediction	Proposed role in S-phase arrest and apoptosis reduction, potentially supporting parasite persistence; Possible minimally invasive diagnostic biomarkers	[125]
*Trypanosoma brucei*	In vitro parasite transcriptomic analysis across life-cycle stages; Parasite mitochondrial transcripts (insect & bloodstream stages)	Multiple mitochondrial circRNA transcripts (Exact number not specified) (Parasite-derived)	RT-PCR + Deep sequencing	No direct functional evidence; Mechanistic hypothesis only	Potential role in mitochondrial gene regulation and RNA stability	[134]

circRNA, circular RNA; GO, gene ontology; KEGG, Kyoto encyclopedia of genes and genomes; MAPK, mitogen-activated protein kinase; miRNA, microRNA; NF-κB, nuclear factor kappa B; RT-PCR, real-time polymerase chain reaction; RNA-seq, RNA sequencing; TGF-β, transforming growth factor beta.

**Table 2:** Key research findings on circRNAs in major helminth parasites.

Parasite	Experimental Model & Sample	No. of Reported circRNAs	Detection Method	Functional Evidence Level	Proposed Biological Role	Ref.
*Schistosoma japonicum*	Parasite transcriptomic profiling; Adult worms	2636 circRNAs (748 in female; 1888 in males) (Parasite-derived)	High-throughput sequencing	Correlation-based; Bioinformatic miRNA prediction	Potential regulatory roles in schistosome development, biological functions and sexual differentiation	[143]
	In vivo murine infection model; Hepatic stellate cells from infected mice	489 differentially expressed (194 up, 295 down) (Host-derived)	High-throughput sequencing + qRT-PCR	Functional validation via knockdown	circGsr–0002 acts as miR-383-3p sponge & involved in schistsome-induced liver fibrosis.	[148]
*Fasciola gigantica*	In vitro stimulation with parasite excretory–secretory products; Goat peripheral blood mononuclear cells	136 differentially expressed (83 up, 53 down) (Host-derived)	RNA seq + qRT-PCR	Correlation-based; pathway prediction only; GO and KEGG analyses	Modulation of immune and metabolic response; proposed role in tolerogenic immune environment	[151]
*Echinococcus granulosus*	In vitro cultures protoscolices + HF from infected sheep; PSC-derived ELVs & hydatid fluid ELVs	Numerous circRNAs (higher abundance in PSC-ELVs vs. HF-ELVs) (Parasite-derived)	High-throughput sequencing	Correlation-based; Bioinformatic circRNA–miRNA–mRNA network prediction	Potential regulation of parasite and host genes at infection interface	[156]
	CE patients vs. healthy liver tissue; Human pericystic liver tissue	343 expressed (177 up, 166 down) (Host-derived)	Microarray analysis + qRT-PCR	Correlation-based	Believed to act as “sponges” for host miRNAs, thereby influencing pathways related to organic cyclic compounds; implicated in the development and progression of CE	[157]
*E. multilocularis*	AE patients vs. healthy controls; Serum exosomes	59 distinct circRNAs (Host-derived)	High-throughput sequencing	Diagnostic correlation; no mechanistic validation yet	Circulating exosomal circRNAs proposed as non-invasive diagnostic biomarkers	[164]
	Experimentally infected mouse model; Murine liver cells	>6000 host circRNAs identified; thousands altered during progression (Host-derived)	RNA-seq	Bioinformatic pathway & miRNA interaction prediction	Modulation of immune response; linked to T cell activation and antigen presentation pathways	[165,166]

AE, alveolar echinococcosis; CE, cystic echinococcosis; circRNA, circular RNA; ELVs, exosome-like vesicles; GO, gene ontology; HF, hydatid fluid; KEGG, Kyoto encyclopedia of genes and genomes; miRNA, microRNA; PSC, protoscolex; qRT-PCR, quantitative real-time polymerase chain reaction; RNA-seq, RNA sequencing.

## Data Availability

The original contributions presented in this review are included in the article.

## Abbreviations

circRNA, Circular RNA; ELVs, exosome-like vesicles; EVs, Extracellular vesicles; GO, Gene Ontology; IRES, Internal Ribosome Entry Site; KEGG, Kyoto Encyclopedia of Genes and Genomes; lncRNAs, long non-coding RNA; miRNA, microRNA; ncRNAs, Non-coding RNAs; qPCR, Quantitative PCR; RBPs, RNA Binding Proteins; RT-PCR, Real-time polymerase chain reaction
